# Cholesterol-induced leucine aminopeptidase 3 (LAP3) upregulation inhibits cell autophagy in pathogenesis of NAFLD

**DOI:** 10.18632/aging.204011

**Published:** 2022-04-11

**Authors:** Lina Feng, Yanping Chen, Ke Xu, Yingchao Li, Farooq Riaz, Kaikai Lu, Qian Chen, Xiaojuan Du, Litao Wu, Dan Cao, Chunyan Li, Shemin Lu, Dongmin Li

**Affiliations:** 1 Department of Biochemistry and Molecular Biology, School of Basic Medical Science, Xi’an Jiaotong University Health Science Center, Xi’an, Shaan Xi 710061, China; 2 Key Laboratory of Environment and Genes Related to Diseases, Xi’an Jiaotong University, Ministry of Education of China, Xi’an, Shaan Xi 710061, China; 3 Department of Infectious Diseases, The Affiliated Hospital of Yan’an University, Yan’an, China; 4 Department of Infectious Diseases, Yan’an Second People’s Hospital, Yan’an, China; 5 Department of Joint Surgery, Xi’an Hong Hui Hospital, Xi’an Jiaotong University Health Science Center, Xi’an, China; 6 Department of Gastroenterology, First Affiliated Hospital of Xi’an Jiaotong University, Xi’an 710061, China

**Keywords:** NAFLD, autophagy, LAP3, cholesterol, biomarker

## Abstract

Objectives: Leucine aminopeptidase 3 (LAP3), an M1 member of leucine aminopeptidase, was reported to be significantly upregulated in serum of nonalcoholic fatty liver disease (NAFLD) patients. However, the underlying mechanisms of LAP3 in NAFLD pathogenesis are still unknown. We aim to investigate the role of LAP3 in NAFLD pathogenesis and explore whether LAP3 has the potential to be a candidate biomarker in serum for NAFLD diagnosis.

Methods: Liver tissues and serum from NASH rats, serum from patients with NAFLD were obtained to evaluate the LAP3 expression. Detection of GSSG/GSH, intracellular reactive oxygen species (ROS), and LC3 expression by elevation/ reduction of LAP3 expression to determine the role of LAP3 in NAFLD pathogenesis. Finally, the correlation analysis was conducted to evaluate the association between LAP3 expression and clinical indexes of NAFLD.

Results: LAP3 expression was upregulated in hepatocytes and serum in E3 rats with NASH after 6-month HFD feeding. Cholesterol (CHO) dramatically upregulated LAP3 in LO2 cells, and then lead to negative regulation of autophagy. Moreover, LAP3 levels were also significantly increased in NAFLD patients compared to healthy controls. Correlation analysis revealed that serum LAP3 levels were positively correlated with TG, γ-glutamyltranspeptidase (GGT), and fasting blood glucose levels, while there was a negative correlation with HDL levels.

Conclusions: The cholesterol-dependent upregulation of LAP3 in hepatocytes plays a critical role in the pathogenesis of NAFLD via inhibiting autophagy. Moreover, LAP3 could serve as a potential novel candidate biomarker for the diagnosis of NAFLD.

## INTRODUCTION

Nonalcoholic fatty liver disease (NAFLD), a chronic liver disorder with hepatic lipid accumulation, is considered a metabolic syndrome that is accompanied by obesity and insulin resistance [1]. It comprises a whole spectrum of fatty liver disease, varying from nonalcoholic fatty liver (NAFL) to nonalcoholic steatohepatitis (NASH), cirrhosis, and hepatocellular carcinoma (HCC) [2, 3]. The latest statistics show that almost 25% world’s population is suffering from NAFLD [4]. Therefore, NAFLD has become a highly prevalent public health concern [5]. Besides that, the development of the NAFLD significantly increases the risks of type 2 diabetes which further increases the risks of cardiovascular and cerebrovascular diseases [6]. Despite the high prevalence of NAFLD, the clinical understanding of NAFLD progression is still insufficient, and there is no universally accepted NAFLD-specific pharmacological treatment. The effective way to solve this health issue is to clarify the etiology and molecular pathogenesis of NAFLD, and determine the potential targets for the early prevention, diagnosis, and treatment of NAFLD [7].

Autophagy and oxidative stress are two essential pathways in NAFLD pathogenesis. Autophagy is a self-degradation pathway that contributes to clearing the cells of all irreversibly oxidized biomolecules (proteins, DNA and lipids) that are oxidized by reactive oxygen species (ROS) and reactive nitrogen (RNS), all of which are important for liver homeostasis in the energy balance [8]. Autophagy not only impacts hepatocytes but also regulates the non-parenchymal cells in the liver, therefore dysfunction of autophagy is associated with many liver diseases such as NAFLD, acute liver injury, chronic alcoholic-related liver injury and hepatocarcinoma [9]. In the setting of NAFLD, obligate-induced oxidative metabolism via increased anabolism could result in oxidative stress and inflammation that reinforce insulin resistance and hepatocellular damage in mice when fed with HFD [10].

Leucine aminopeptidases (LAPs), cell maintenance enzymes that can catalyze the leucine residues cleaved from the N-amino termini, play a variety of functions in mammals and plants [11]. LAP3, one of the important M1 members of LAPs, serves multifunctional roles in tumor metastasis, such as promoted cell proliferation and migration in glioma tumors [12, 13], advanced malignant development of human ESCC [14, 15]. In breast cancer, overexpression of LAP3 down-regulates the phosphorylation of Hsp27 and upregulates the expression of fascin, Akt phosphorylation, and matrix metalloproteinase-2/9(MMP2/9) [16]. Many studies have proved that MMP2/9 and Akt are involved in the pathogenesis and progression of NAFLD [17–19]. In addition, LAP3 is dramatically upregulated in the disease-related to hyperinflammation and is considered as a potential anti-inflammatory drug target [20]. What’s more, a recent proteomics-based study has revealed that LAP3 is significantly elevated in the plasma of NAFLD patients [21]. To the best of our knowledge, the regulatory roles of LAP3 in metabolic disorders during the progression of NAFLD have not been reported. Therefore, we aimed to study the role of LAP3 in NAFLD pathogenesis.

In this study, we first demonstrated that LAP3 expression was increased in the hepatocytes and serum from HFD-induced NASH rats. CHO increased LAP3 expression and then upregulation of LAP3 took part in NAFLD pathogenesis through inhibiting cell autophagy rather than oxidative stress. Lastly, we validated that LAP3 expression was also higher in serum of NAFLD patients. Importantly, our study showed that the elevation of LAP3 in serum is positively correlated with fasting blood glucose, GGT (γ-glutamyltranspeptidase), and TG (triglyceride), whereas negatively correlated with LDL. Collectively, our results suggest that LAP3 could play an important role in the pathogenesis of NAFLD.

## RESULTS

### LAP3 is increased both in serum and in hepatocytes in HFD-induced NASH rats

**Figure 1:**
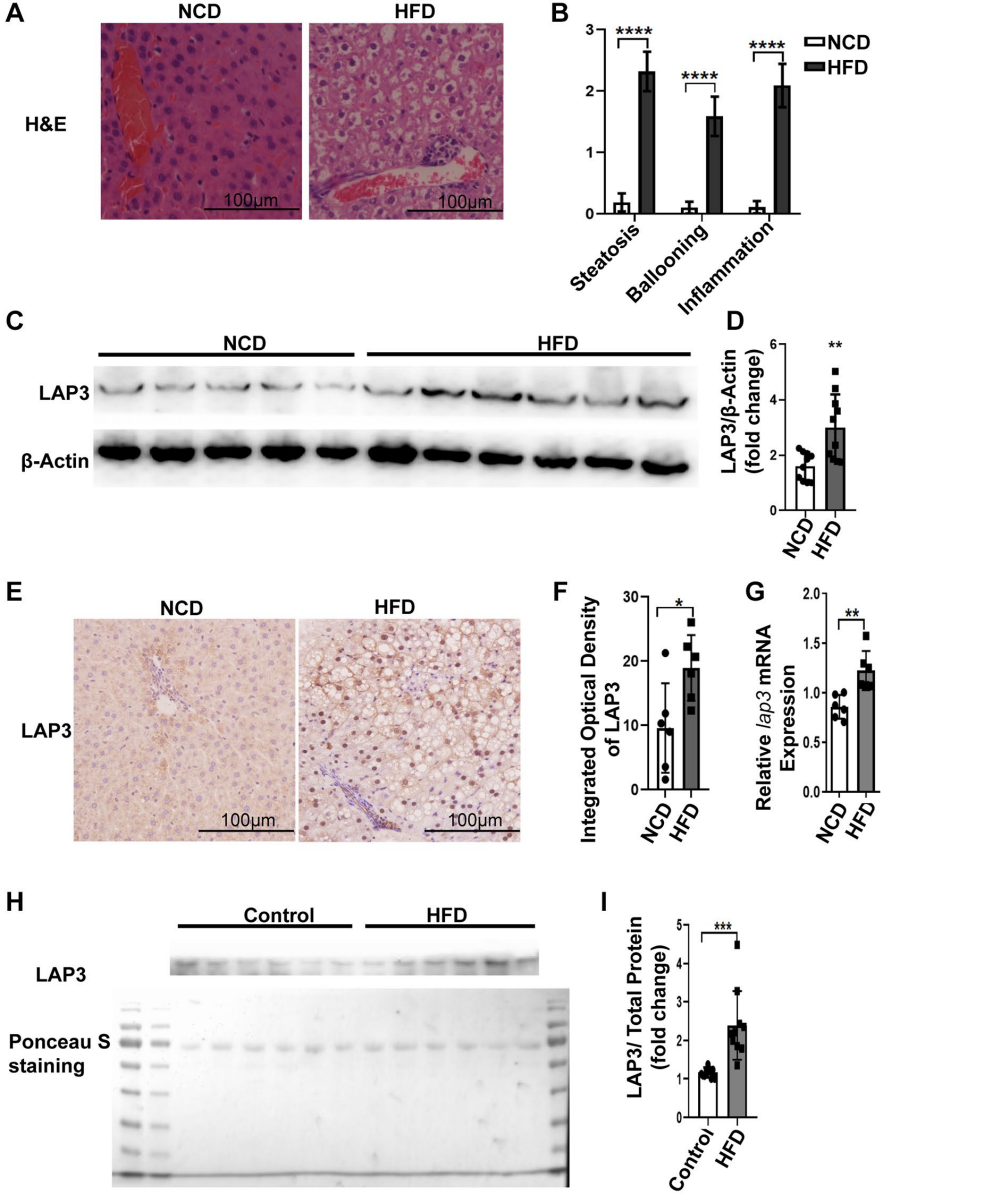
**LAP3 was upregulated in 6-month HFD-induced E3 rats with NASH.** (**A**) H&E staining of liver specimen for each animal group. (**B**) The score for hepatic steatosis, hepatocytes ballooning and lobular inflammation were analyzed (*n* = 10 per group). (**C** and **D**) The protein expression of LAP3 in livers of 6-month HFD induced NASH E3 rats were evaluated by Western blotting (**C**) and normalized by β-actin (**D**) (*n* = 11 for each group). (**E**) Increased LAP3 is predominantly expressed in hepatocyte nucleus in 6-month HFD induced NASH E3 rats through Immunohistochemistry (*n* = 10 per group). (**F**) Statistics for immunohistochemistry result by Image pro plus. (**G**) *lap3* mRNA expression in livers of 6-month HFD induced NASH E3 rats was detected by RT-qPCR (*n* = 10 per group). (**H** and **I**) Serum LAP3 content in 6-month HFD induced NASH E3 rats was detected by western blotting (**F**) and normalized by serum total protein (**G**) (*n* = 10 per group). Data are expressed as means ± SD from three independent experiments. ^*^p < 0.05; ^**^p < 0.01; ^***^p < 0.001. Abbreviations: HFD-NASH: high-fat-diet-induced nonalcoholic steatohepatitis; RT-qPCR: quantitative Realtime PCR.

Emerging studies have reported that LAP3 was involved in different liver diseases [15, 21]. To explore the involvement of LAP3 in NASH, we firstly examined animal model. As representative images of H&E staining showed that rats fed with HFD developed severe steatosis, ballooning, and inflammation infiltrates, compared to NCD group. These all indicated that our NASH animal model was successful (Figure 1A and 1B). To further explore the expression of LAP3 in the liver and serum from E3 rats with NASH after 6-month HFD feeding, we first detected the LAP3 expression in protein level and mRNA level in the liver tissue from HFD and NCD group by western blotting and RT-qPCR, respectively. As the results showed that LAP3 was significantly elevated in the liver in the HFD group at both protein level (Figure 1C and 1D) and mRNA level (Figure 1G), compared to the normal control diet (NCD) group. To further determine the location of upregulated LAP3 in the liver, we performed LAP3 immunohistochemistry (IHC) staining and the result demonstrated that LAP3 is both in the nucleus and cytoplasm of hepatocytes. Moreover, elevated LAP3 is predominantly localized in the nucleus in the HFD group (Figure 1E and 1F). Since LAP3 can be detected in serum, we next measured LAP3 expression in serum by western blotting and the result exhibited that LAP3 was significantly increased in serum of HFD-induced NASH animal model (Figure 1H and 1I).

### Cholesterol results in LAP3 elevation in LO2 cells

**Figure 2:**
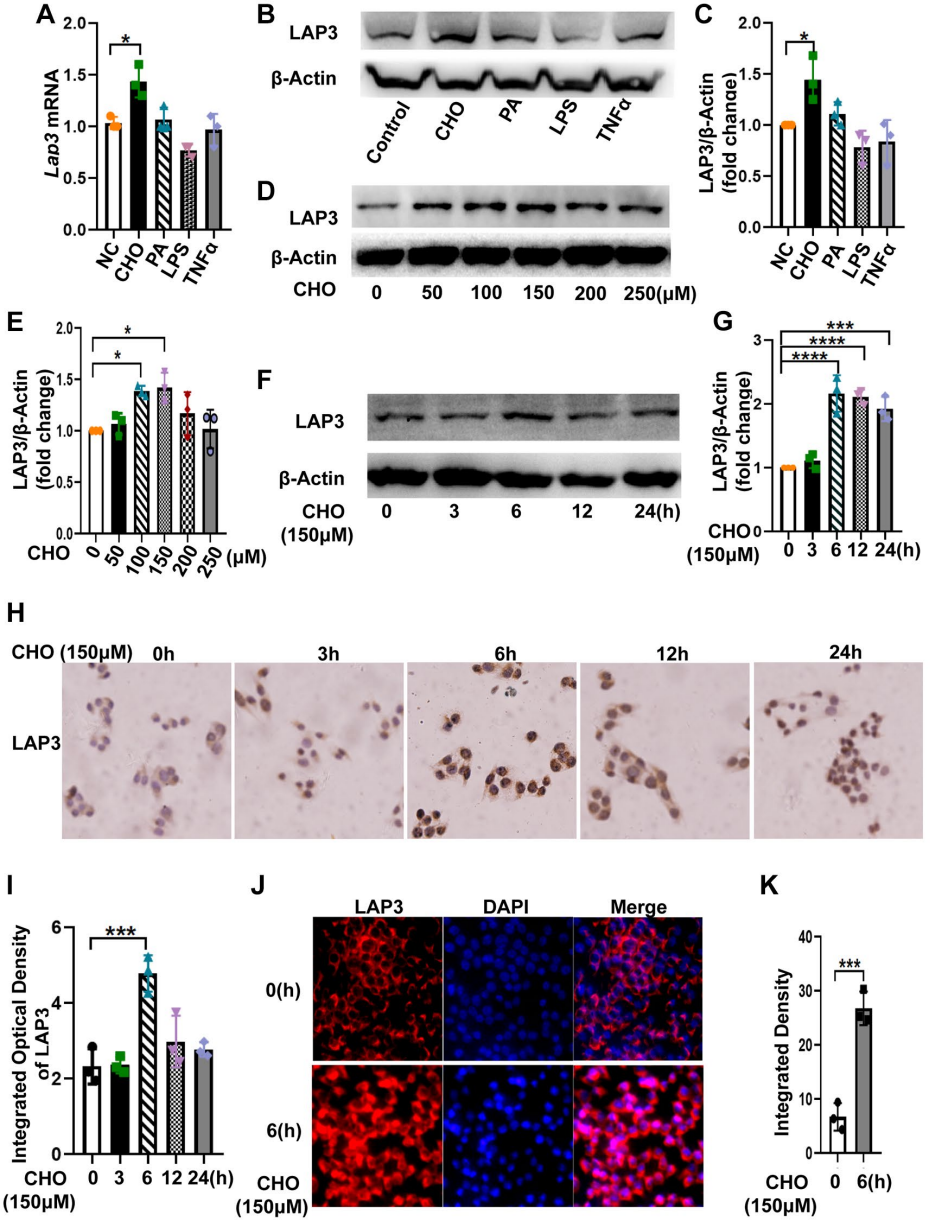
**Cholesterol increased LAP3 expression, which is mainly localized in the nucleus of LO2 cells.** (**A**–**C**) After LO2 cell line treatment with 150 μM CHO, 200 μM PA, 5 μM LPS or 10 ng/ml TNF-α for 24 h, the mRNA expression of lap3 was detected by RT-qPCR (**A**), the protein expression of LAP3 was detected by western blotting (**B**) and normalized by β-actin (**C**). (**D** and **E**) LO2 cell line treated using different concentration 0, 50,100, 150 and 250 μM of CHO for 24 h respectively and the protein expression of LAP3 was detected by western blotting (**D**) and normalized by β-actin (**E**). (**F**–**J**) LO2 cell line treated with 150 μM of CHO for the indicated time points, the protein expression of LAP3 was detected by western blotting (**F**) and normalized by β-actin (**G**), and further validated by IHC (**H**) and IF (**J**), respectively. (**I** and **K**) Statistics for IHC and IF by Image pro plus. Data are expressed as means ± SD from three independent experiments. ^*^p < 0.05; ^**^p < 0.01; ^***^p < 0.001. An unpaired *t*-test and one-way ANOVA were applied to determine the statistical significance. Abbreviations: CHO: cholesterol; PA: palmitic acid; IHC: immunohistochemistry; IF: immunofluorescence.

To further explore the factors that contribute to the high expression of LAP3 in hepatocytes of HFD-induced NASH rat model, we treated LO2 cells with 150 μM cholesterol (CHO), 200 μM palmate acid (PA), 5 μM LPS, and 10 ng/ml TNF-α for 24 hours, respectively. Results from RT-qPCR and western blotting together showed that only CHO caused an upregulation of LAP3, whereas LPS significantly decreased LAP3 expression. PA and TNFα treatment did not affect LAP3 expression in the LO2 cells (Figure 2A–2C). Subsequently, we incubated LO2 cells with 0 μM, 100 μM, 150 μM, 200 μM, 250 μM and 300 μM of cholesterol for 24 h. Western blotting results showed that the optimum concentration of CHO on induction of LAP3 in LO2 cells was 150 μM (Figure 2D and 2E). We then treated LO2 cells with 150 μM CHO at different time points, and the results showed that the expression of LAP3 in the LO2 cells reached the maximum after 6 hours of CHO treatment (Figure 2F and 2G). To determine the localization of LAP3 in CHO-treated LO2 cells, timepoint LAP3-IHC (Figure 2H and 2I) and LAP3- IF (Figure 2J and 2K) staining were conducted and consistently in our animal model, the results both showed a significant nucleus upregulation of LAP3.

### LAP3 upregulation triggered by CHO does not participate in the regulation of oxidative stress in LO2 cells

**Figure 3:**
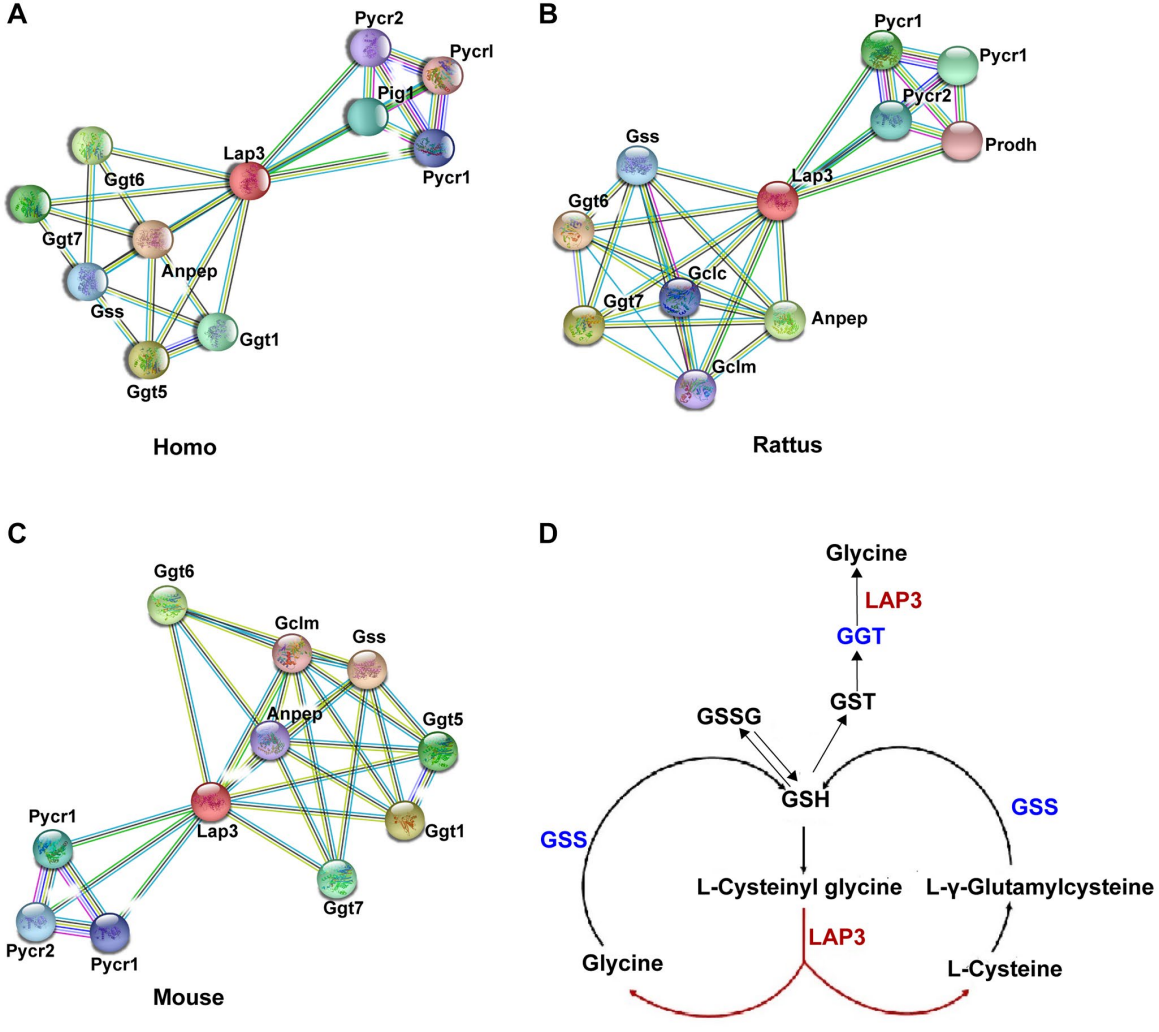
**Bioinformatic analysis indicated that LAP3 played a significant role in GSH metabolism.** (**A**–**C**) Analysis of top ten proteins that interact with LAP3 in human, rat, and mouse using the STRING database. (**D**) Screened pathway showing the involvement of LAP3 in GSH metabolism using KEGG database.

To better clarify the underlying molecular mechanism of LAP3 in NAFLD pathogenesis, we utilized the STRING database (https://string-db.org/) to analyze which proteins could interact with LAP3 in humans, rats, and mice, respectively. Compared to the top ten proteins in three species, we found that most of these proteins were involved in the oxidative stress pathway, such as Ggt, Gclm, Gss, and Anpep (Figure 3A–3C). At the same time, we investigated the possible pathways of LAP3 using KEGG: Kyoto Encyclopedia of Genes and Genomes database (https://www.genome.jp/kegg/), and found that LAP3 was mainly involved in the three pathways: 1) Arginine and proline metabolism. 2) Glutathione metabolism. 3) Metabolic pathway. Combined the analysis of STRING and KEGG indicated that LAP3 may take part in the GSH pathway. It has been widely acknowledged that oxidative stress played a central role in the pathogenesis of NAFLD due to the onset of lipid droplet accumulation in hepatocytes in NAFLD being closely associated with the change of the redox status [17]. Therefore, we first examined whether LAP3 could regulate glutathione metabolism (Figure 3D).

**Figure 4:**
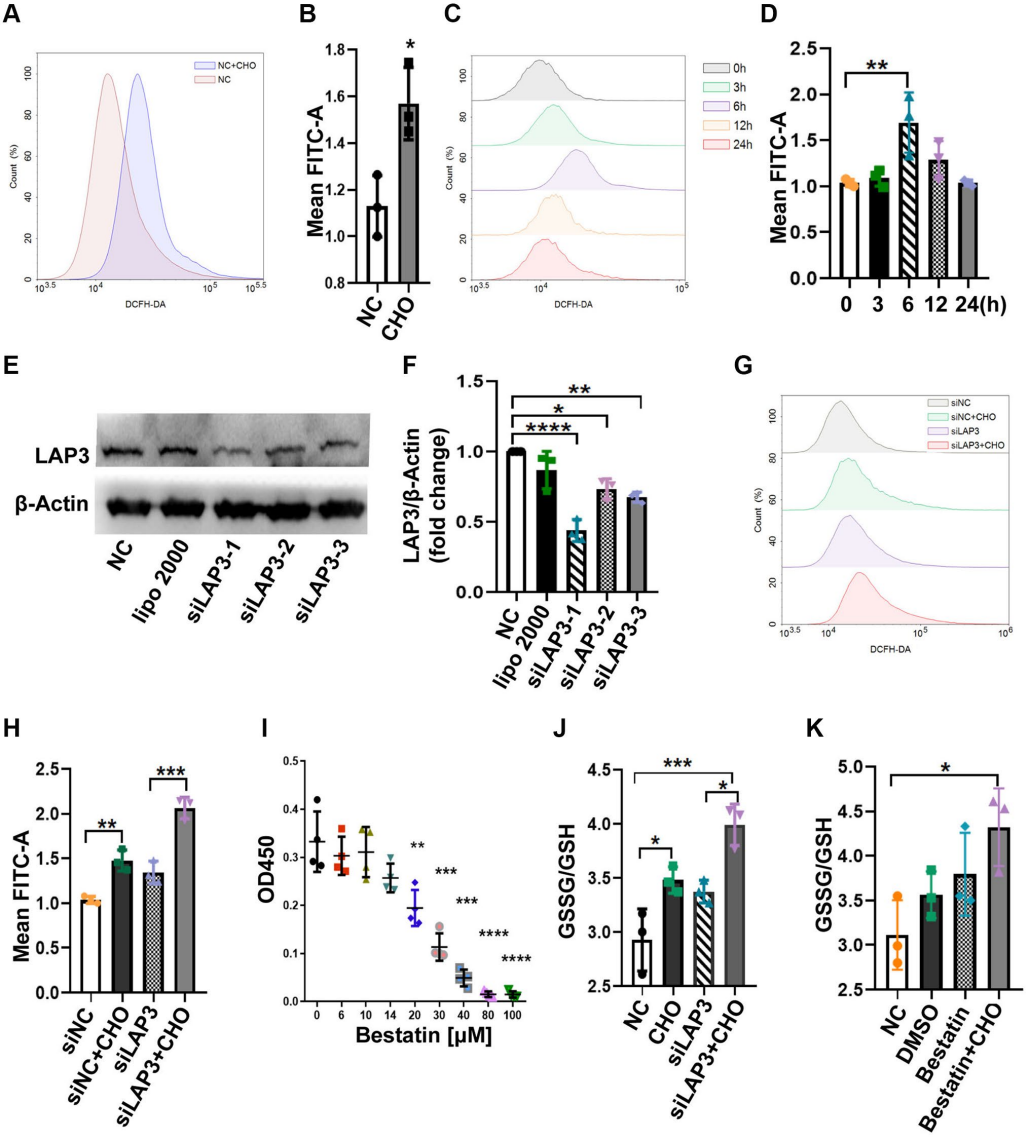
**LAP3 upregulation triggered by CHO does not participate in regulation of oxidative stress in LO2 cells.** (**A** and **B**) Evaluation of the DCFH-DA fluorescence intensity for 150 μM CHO treatment LO2 cell line at 6 h by flow cytometry (**A**) and normalized with NC group (**B**). (**C** and **D**) Evaluation of DCFH-DA fluorescence intensity at indicated time points in LO2 cell line treated with 150 μM CHO (**C**) and normalized with 0 h data (**D**). (**E** and **F**) Screening of siLAP3 to knock-down LAP3 expression by western blotting (**E**) and normalized with β-Actin (**F**). (**G** and **H**) Detection of DCFH-DA for the generation of ROS in LO2 cells after the intervention of lap3 expression by siLAP3 (**G**) and normalized with vehicle (**H**). (**I**) Evaluation of cell viability after treatment of LO2 cell with indicated concentration bestatin, a LAP3 natural inhibitor, for 6 h. (**J** and **K**) Determination of GSSG/GSH in LO2 cell line treated with 150 μM CHO and siLAP3-1 (**J**) or 14 μM Bestatin (**K**) for 6 h. Data are expressed as means ± SD from three independent experiments. An unpaired *t*-test and one-way ANOVA were applied to determine the statistical significance with Graphpad Prism 8, ^*^p < 0.05; ^**^p < 0.01; ^***^p < 0.001. Abbreviations: CHO: cholesterol; GSH: L-Glutathione; GSSG: glutathione (oxidized form).

We compared the reactive oxygen species (ROS) levels between CHO-treated cells and control groups in LO2 cells by flow cytometry. The results showed that CHO-treated LO2 cells upregulated the ROS levels compared to the normal control (NC) group (Figure 4A and 4B). Moreover, the intracellular ROS levels have reached the maximum after 6 hours of 150 μM CHO treatment which was consistent with LAP3 expression patterns (Figure 4C and 4D). The above results suggested that CHO upregulated LAP3 was associated with increased intracellular ROS. To further confirm the association between LAP3 and ROS, LO2 cells were treated with three synthesized LAP3 siRNAs to knock down its expression. The results showed that siLAP3-1 showed higher efficiency in knocking down the expression of LAP3 (Figure 4E and 4F). The intracellular ROS levels were still elevated independently of downregulation of LAP3 by siLAP3-1 in LO2 cells (Figure 4G and 4H). To confirm the above results, we reduced the LAP3 expressions by its natural inhibitor bestatin. And the result displayed that 14 μM bestatin did not affect LO2 cells viability as determined by the CCK-8 assay (Figure 4I). Then we tested the GSSG/GSH in an increase or the decrease LAP3 expression by CHO or siLAP3, respectively, and results together showed that ROS significantly increased dependently on the CHO stimulation (Figure 4J). In addition, we examined GSSG/GSH in the CHO-treated LO2 cells with bestatin, and we obtained similar results with LAP3 silence (Figure 4J and 4K). Overall, these results demonstrated that CHO-induced elevation of LAP3 expression does not participate in the regulation of oxidative stress.

### Upregulation of LAP3 by cholesterol inhibits autophagy in LO2 cells

**Figure 5:**
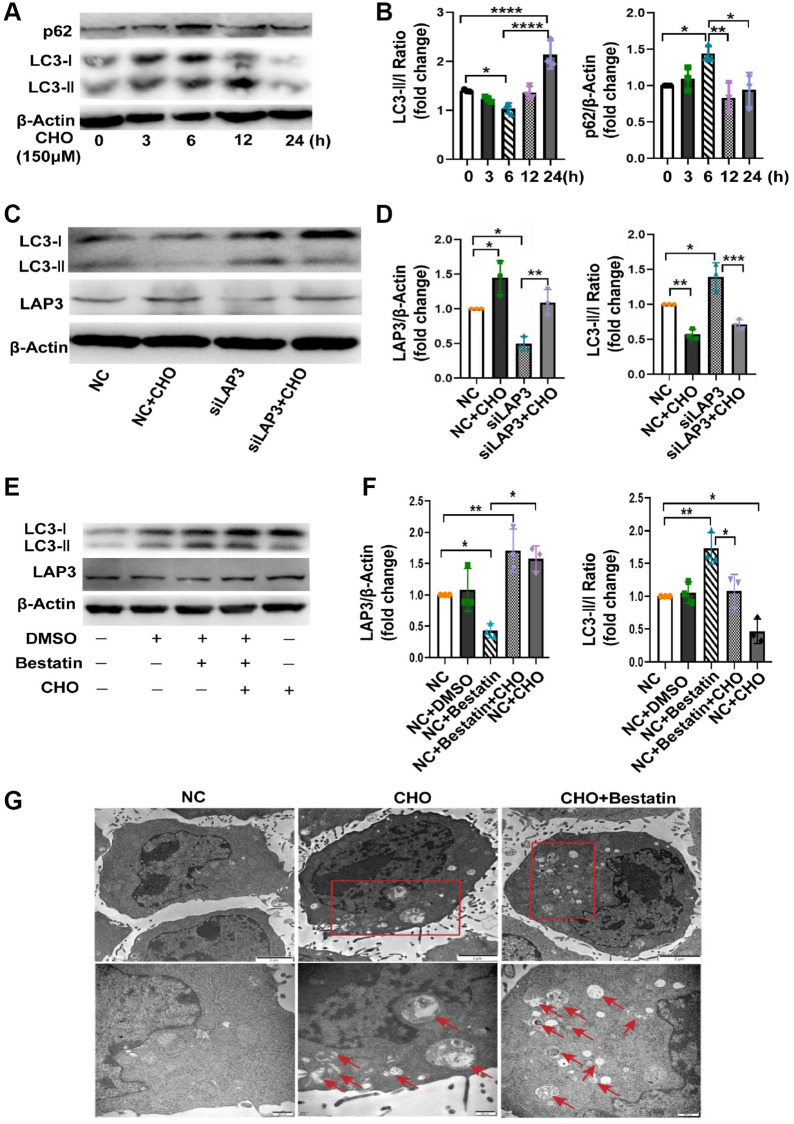
**Upregulation of LAP3 by cholesterol inhibits autophagy in LO2 cells.** (**A** and **B**) Representative western blotting detailing autophagic flux in CHO-treated LO2 cells (**A**) and quantitative analysis (**B**). (**C** and **D**) Evaluation of LAP3 and autophagy marker LC3 II and LC3 I expression after treatment of LO2 cells with 150 μM CHO or siLAP3 at 6 h by western blotting (**C**) and quantitative analysis (**D**). (**E** and **F**) Evaluation of LAP3 and LC3 II and LC3 I expression after treatment LO2 cell with 150 μM CHO or 14 μM bestatin at 6 h by western blotting (**E**) and quantitative analysis (**F**). (**G**) LO2 cells were treated with 150 μM CHO or 14 μM Bestatin for 6 h, the formation of autophagic vacuoles (→) was examined by transmission electron microscopy analysis. Data are expressed as means ± SD from three independent experiments. ^*^p < 0.05; ^**^p < 0.01; ^***^p < 0.001. A one-way ANOVA test was performed to determine the statistical significance. Abbreviations: CHO: cholesterol; DMSO: dimethyl sulfoxide.

Numerous studies have proved that CHO take an essential role in autophagy [22, 23]. Meanwhile, autophagy plays a remarkable role in NAFLD progression [24]. Thereafter, we attempted to explore whether upregulation of LAP3, triggered by CHO, was involved in the regulation of autophagy. We first investigated the autophagic flux by treating LO2 cells with CHO at different timepoint. The western blotting results showed that autophagy flux was gradually decreased during 0–6 h after CHO treatment, then elevated in 12 h and 24 h. And p62, a substrate for autophagy autophagolysosomal proteolysis, was found to be increased during 0–6 h, decreased from 12 h (Figure 5A and 5B). Meanwhile upregulation of LAP3 after treatment with 150 μM CHO for 6 hours reduced the autophagy marker LC3 II/I ratio, while knockdown the expression of LAP3 by siLAP3 increased LC3 II/I ratio in LO2 cells (Figure 5C and 5D). Similar observations were found in LO2 cells treatment with LAP3 inhibitor, bestatin (Figure 5E and 5F). To further confirm these observations, we analyzed the LO2 cells treated with CHO or bestatin with TEM, the gold standard method for autophagy. The results showed that double-membrane autophagosomes were seen in cells treated with CHO and bestatin, compared with the control group. And the number of autophagosomes was dramatically increased after reduction of LAP3 expression by bestatin, compared with upregulation of LAP3 by CHO (Figure 5G). Collectively, all these results demonstrated that CHO-dependent upregulation of LAP3 expression inhibits autophagy in the LO2 cells.

### LAP3 is upregulated in serum from NAFLD patients and correlated with GLU, GGT, TG, and HDL

To further investigate whether LAP3 could be a candidate biomarker for clinical detection of NAFLD, the serum from 40 NAFLD patients and 40 healthy subjects was obtained. There were increased levels of liver enzymes such as ALT, AST, ALP, GGT in serum from the NAFLD group and also higher TC and TG levels, while there was a decrease in ALB and HDL compared with the Control group (Table 1).

**Table 1:** Baseline characteristics of the serum participants.

	**Healthy**	**NAFLD**	***p*-value**
Gender (Female/Male)	23/17	15/25	0.8440
ALB (g/L)	47.0921 ± 4.2463	43.7455 ± 7.4785	0.0232
GLOB (g/L)	28.5921 ± 3.7687	28.7636 ± 6.0756	0.8868
ALT (U/L)	18.475 ± 9.0083	46.9697 ± 48.3193	0.0006
AST (U/L)	21.275 ± 4.5661	34.3824 ± 29.9539	0.0088
ALP (U/L)	65.1316 ± 18.674	83.5807 ± 32.6267	0.005
GGT (U/L)	16.2368 ± 8.6311	52.2258 ± 52.3263	<0.0001
TC (mmol/L)	4.0688 ± 0.5418	4.6415 ± 0.974	0.0024
TG (mmol/L)	1.1748 ± 0.2467	2.2413 ± 1.9317	<0.0001
HDL-C (mmol/L)	1.2587 ± 0.1904	1.1139 ± 0.3289	0.0262
LDL-C (mmol/L)	2.4581 ± 0.5027	2.6868 ± 0.8431	0.1821

Values are present as mean ± SD. Abbreviations: ALT: alanine aminotransferase; AST: aspartate aminotransferase; GLOB: globulin; ALP: alkaline phosphatase; GGT: γ-glutamyltranspeptidase; TC: total cholesterol; TG: total triglyceride; HDL-C: high-density lipoproteins; LDL-C: low-density lipoproteins.

**Figure 6:**
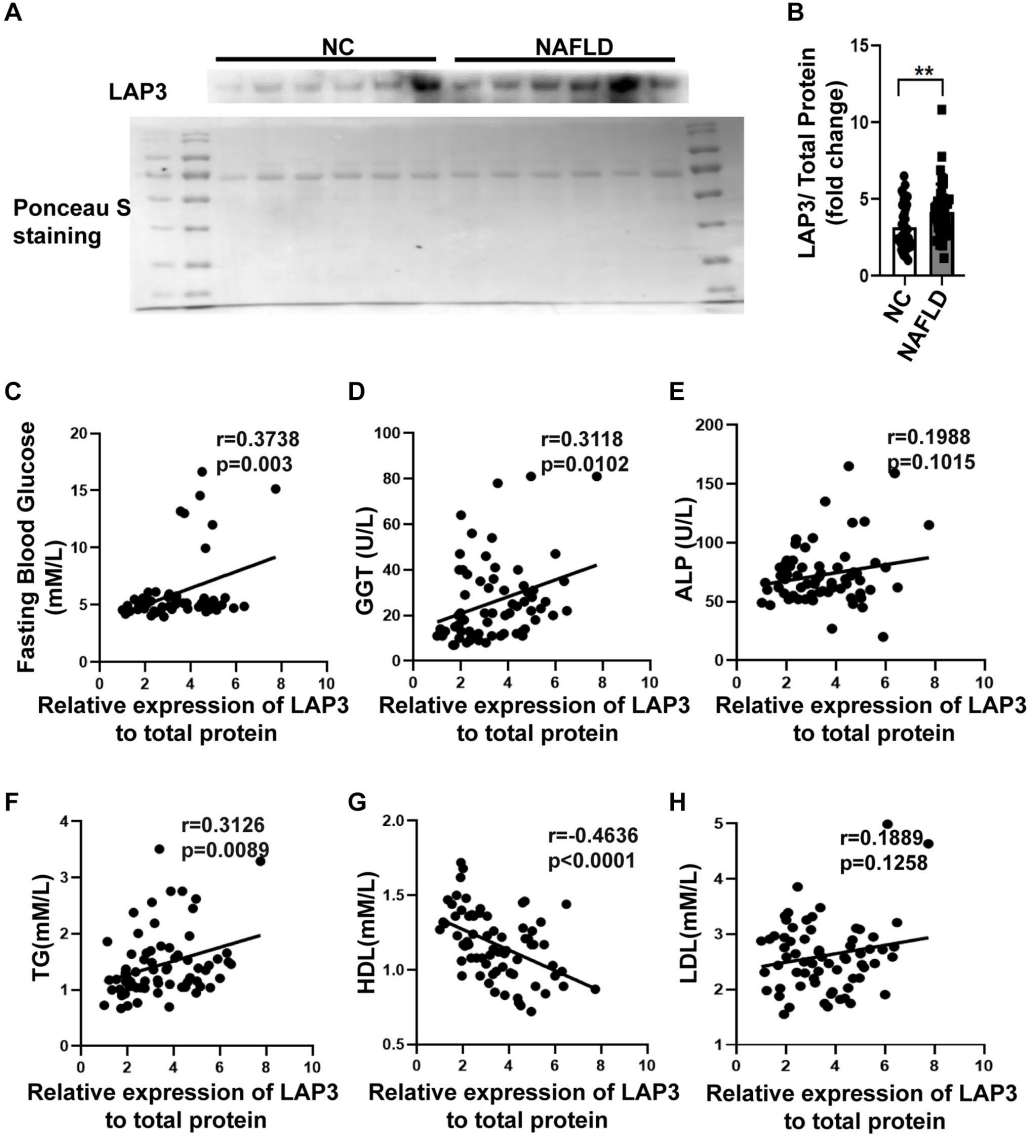
**LAP3 was upregulated in the plasma of NAFLD patients.** (**A** and **B**) The LAP3 expression in plasma of NAFLD patients and normal people was detected by western blotting (**A**) and normalized by serum total protein (**B**). (**C**–**H**) Correlation analysis between LAP3 and clinical indexes of NAFLD by Graph prism 8.0. Data were presented as means ± SD. ^*^p < 0.05; ^**^p < 0.01; ^***^p < 0.001. A two-tail *t*-test was performed to determine the statistical significance. Abbreviations: NAFLD: Nonalcoholic fatty liver disease; NC: normal healthy control; GGT: γ-glutamyltranspeptidase; ALP: alkaline phosphatase; TG: triglyceride; HDL: high-density lipoprotein; LDL: low-density lipoprotein.

Western blotting was performed to evaluate the LAP3 expression in serum of NAFLD patients and NC. The result displayed that LAP3 was increased in serum of NAFLD patients compared with the NC group (Figure 6A and 6B). Additionally, we conducted a correlation analysis between LAP3 levels in serum and NAFLD clinical indexes. The results showed that LAP3 was positively correlated with fasting blood glucose (*p* = 0.003, r = 0.3738) and GGT (*p* = 0.0102, r = 0.3118) (Figure 6C and 6D), while it had no correlation with ALP (Figure 6E). Moreover, LAP3 levels were positively correlated with TG levels (*p* = 0.0089, r = 0.3126) and negatively correlated with HDL levels (*p* < 0.0001, r = −0.4636) (Figure 6F and 6G), while there was no relevance to LDL level (Figure 6H).

## DISCUSSION

**Figure 7:**
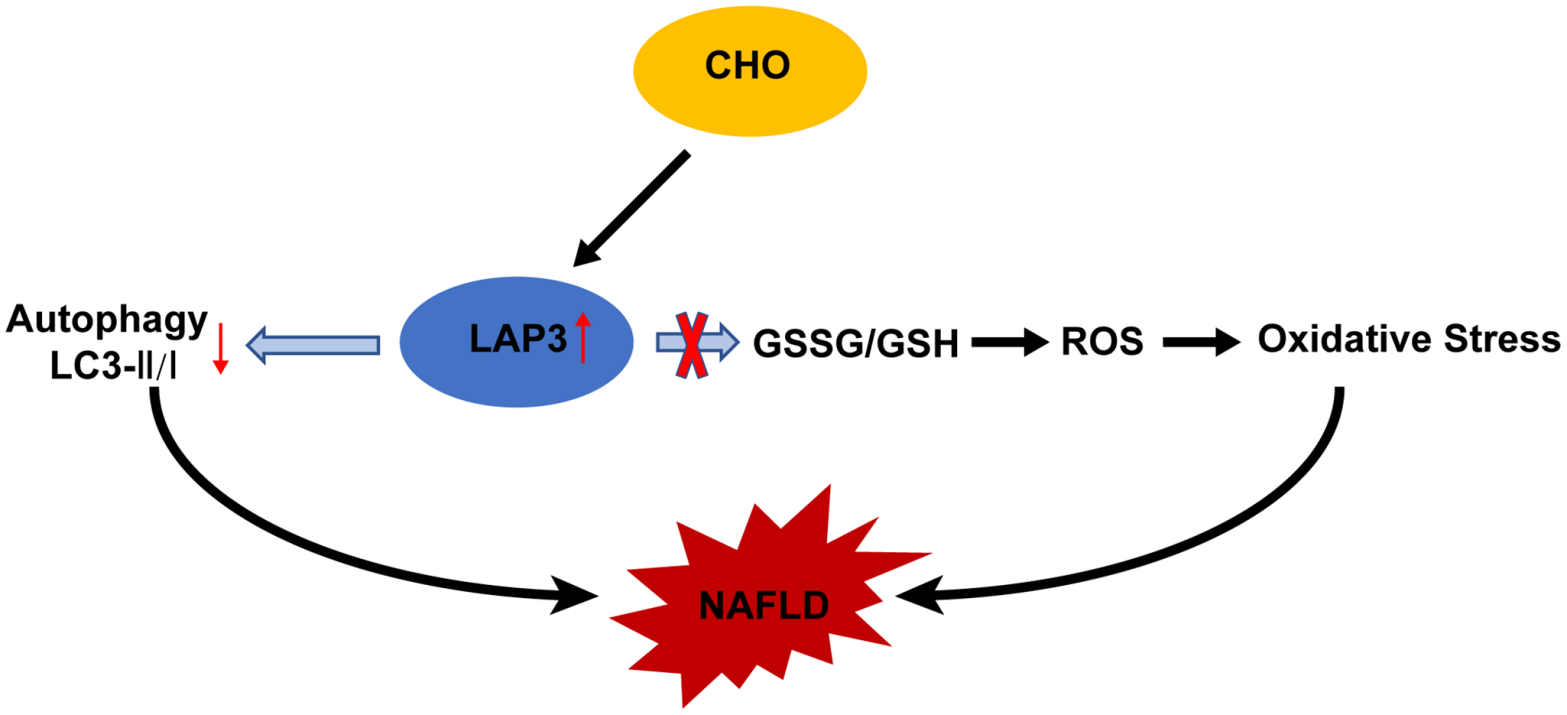
**Molecular mechanism of cholesterol-induced LAP3 upregulation in hepatocytes participated NAFLD pathogenesis.** Abbreviations: CHO: cholesterol; NAFLD: nonalcoholic fatty liver disease; ROS: reactive oxygen species; LAP3: leucine aminopeptidase 3.

In this study, we demonstrated that LAP3 played a critical role in the pathogenesis of NAFLD. The expression of LAP3 was upregulated not only in hepatocytes of NASH rats but also in serum of NAFLD patients and HFD-induced E3 rats with NASH. And elevated or decreased LAP3 expression by CHO or siLAP3, bestatin respectively, showed a significant inhibition or exacerbation of autophagy, but not oxidative stress in LO2 cells (Figure 7). Moreover, our correlation analysis pointed out that LAP3 expression in serum was positively correlated with fasting blood glucose, GGT, and TC, while negatively correlated with HDL.

NAFLD, the most common cause of liver fibrosis and HCC, is highly prevalent and one of the leading health problems worldwide [5]. NAFLD is diagnostic based on conventional ultrasonography to ensure steatosis, clinico-laboratory assessment to rule out other competition disease, serum biomarkers of fibrosis, and/or ultrasound-based elastography to stratify risks three-step approach has been established [25]. However, liver biopsy, an invasive, expensive, and time-consuming, related to pain or discomfort method, is still the gold standard [26]. These all emphasize developing potential diagnostic markers and new therapies for NAFLD diagnosis and treatment, respectively [27]. LAP3 was originally served as an important maintenance enzyme that can cleave N-terminal residues from proteins and peptides [11]. Previous studies have reported that LAP3 exerted harmful roles in the pathogenesis and progression of many tumor diseases [12, 14–16]. Recently, one proteomics-based study reported that elevation of LAP3 in serum could be a predictor for early-stage of NAFLD [21]. However, the causal mechanism of LAP3 in NAFLD pathogenesis has not been elucidated. Here, we showed that LAP3 is significantly upregulated in hepatocytes and serum from rats with NASH and serum from the patients with NAFLD. This study was the first to obtain insights into the relationship between LAP3 and NASH induced by HFD. Meanwhile, NAFLD is characterized by excessive hepatic lipid accumulation with significant elevation of TC, TG, LDL, LDL/HDL, TG/HDL, TC/HDL, and lower HDL, accompanied by obesity, type 2 diabetes, compared to subjects without NAFLD [23, 26, 28]. Consistently, we firstly found that LAP3 expression was positively correlated with GGT, fasting blood glucose, while negatively correlated with HDL levels. AST exists both in the cytosol and mitochondria of the hepatic cell, while ALT only presents in the cytosol. Whenever there is hepatocyte injury, AST and ALT are transfused into the blood [29]. ALP and GGT are the enzymes that are related to the biliary ducts of the liver. GGT is specifically expressed by the liver and is considered a sensitive marker for cholestatic damage compared to ALP when its value increases [29, 30]. These results provide experimental evidence that LAP3 might be a novel promising biomarker candidate in serum for NAFLD diagnosis.

Autophagy and oxidative stress are the two important pathways involved in NAFLD [28, 31–33]. Accumulated studies demonstrated a crosstalk between autophagy and oxidative stress since they share some common regulators, such as CHO [23, 34] and Nrf2 [35]. Free cholesterol accumulation in hepatocytes, which resulted from a high cholesterol diet or a western-style diet, can lead to NAFLD, NASH, and hepatic fibrosis in mice. It has been reported that autophagy is significantly impaired in 16 weeks HFD-induced NASH model [22]. In NAFLD, declined autophagy in hepatocytes leads to hepatotoxicity and accumulation of lipid droplets, thus contributing to hepatocyte death [36]. Here we found that CHO in HFD mainly elevated LAP3 expression in the nucleus of LO2 cells. And CHO treatment also increased the intracellular ROS and GSSG/GSH, while knockdown of LAP3 expression in LO2 cells by siRNA or LAP3 inhibitor bestatin, did not affect the ROS and GSSG/GSH. These data indicated that the increase of oxidative stress was due to CHO, but not the elevation of LAP3. However, upregulation of LAP3 by CHO decreased the LC3II/I, while the autophagy was increased after we reduced LAP3 via siRNA or bestatin in LO2 cells. All these results indicated that LAP3 negatively regulated autophagy in LO2 cells. Taken together, our results indicated that CHO increases LAP3 expression in hepatocytes and upregulation of LAP3 takes part in NAFLD pathogenesis by inhibiting cell autophagy, rather than oxidative stress. To the best of our knowledge, our study is the first to assess the mechanism of LAP3 on HFD-induced NAFLD progression. LAP3 was present in the cytoplasm in the previous studies [12, 16], we discovered that LAP3 was mainly increased in the nucleus of hepatocytes in both the HFD-induced NASH rats and LO2 cells treatment with CHO. It may be due to the different cells and different tissues.

However, we did not identify the regulatory mechanism of LAP3 negatively regulated autophagy in hepatocytes in this article. Enlarging the number of serum and liver biopsies of NAFLD patients to validate whether LAP3 can be a biomarker of NAFLD diagnosis, and exploring the roles of LAP3 in inflammation and lipid accumulation are the goals in the future.

## CONCLUSIONS

In summary, LAP3 plays a vital role in the pathogenesis of NAFLD, and it has the potential to represent a candidate biomarker for clinical NAFLD diagnosis.

## MATERIALS AND METHODS

### HFD-induced NASH animal model

There were well-established E3 rats with NASH after 6-month high-fat diet (HFD) feeding models. Briefly, a total of 24 sex-matched E3 rats (originally obtained from the Section of Medical Inflammation Research, Lund University, Sweden) were randomly divided into the normal control diet (NCD) group and the HFD group (male = 6, female = 6 per group) at 8–12 weeks of age. Then the control group rats were fed with a normal diet (10% of total energy as fat), and the HFD group rats were fed with HFD (36% of total energy as fat) as previously described [37]. After 6 months old, the rats were sacrificed, serum and liver tissues were collected and used for subsequent experiments. Biopsy-proven NC group and HFD group as described in our previous study [38]. This project was approved by the Institutional Animal Ethics Committee of Xi’an Jiaotong University Health Science Center (NO. XJ2013086). Detail grading methods for NAFLD were as previously described [39, 40], and summarized in Table 2.

**Table 2:** Nonalcoholic steatohepatitis activity score.

**Score**	**Hepatic Steatosis** **(0–3)**	**Hepatocyte Ballooning** **(0–2)**	**Lobular Inflammation** **(0–3)**
0	<5%	None	No foci
1	5–33%	A few	1–2 foci per 200× field
2	33–66%	many cells	2–4 foci per 200× field
3	>66%	/	>4 foci per 200× field

### Cell culture

The human normal hepatocyte cell line (LO2) was cultured using Roswell Park Memorial Institute-1640 medium (RPMI 1640) (Hyclone, South Logan, UT, USA) supplemented with 10% fetal bovine serum (FBS) (Gibco, Gaithersburg, MD, USA), and maintained in 5% CO_2_ incubator at 37°C. LO2 cells were treated with palmitic acid (Sigma-Aldrich, St. Louis, MO, USA), CHO (Sigma-Aldrich, St. Louis, MO, USA), LPS (Sigma-Aldrich, St. Louis, MO, USA), or (Proteintech, Wuhan, China), TNF-a (Sigma-Aldrich, St. Louis, MO, USA) to screen factors which induced LAP3 upregulation.

### Western blotting

To detect protein expression, total protein from the harvested LO2 cells and liver tissues were extracted by using RIPA buffer following instruction (Beyotime Biotechnology, Shanghai, China) with a 1% protease inhibitor cocktail (MCE, Monmouth Junction, NJ, USA). The concentration of protein was evaluated with BCA protein assay kit (Thermo Fisher Scientific, USA). Proteins were separated on 12% SDS-PAGE gel and transferred onto polyvinylidene fluoride (PVDF) membranes (Bio-Rad, Hercules, CA, USA). Then PVDF membranes were blocked with 5% non-fat milk in TBST for 2 hours at room temperature, and the membranes were incubated with primary antibodies LAP3 (ABclonal Technology, Wuhan, China, 1:1000), β-actin (Abcam, Cambridge, MA, USA, 1:2500), and LC3 (CST, USA, 1:500) at 4°C overnight. Membranes were washed with TBST (contained 0.15% Tween 20), and then incubated with the secondary goat anti-rat (Zhuangzhi; Xi’an; China; 1:5000), or goat anti-mouse (Zhuangzhi; Xi’an; China; 1:5000) antibody for 2 h at room temperature. Membranes were visualized with Gene Gnome XRQ System. Relative quantification of protein levels was achieved by measuring the intensity of the bands with the Fusion software, normalized with β-actin.

### Quantitative real-time PCR (RT-qPCR)

To evaluate gene expression at the mRNA level, real-time quantitative PCR (RT-qPCR) was performed. Total RNA from LO2 cells and E3 rat liver tissues was extracted using TRIzol^®^ (Invitrogen, USA). 5 μg total RNA per sample was reverse transcribed by the RevertAid First Strand cDNA Synthesis Kit (Thermo Fisher Scientific; USA) after the concentration and the quality was determined by Nanodrop. RT-qPCR was performed with SYBR^®^ Premix Ex Taq™ II (Rox; Roche, Basel, Switzerland) in the Agilent Mx3005P systems. The 2^−ΔΔCt^ method was used to determine the relative quantitative gene expression levels, normalized by β-actin. ALL the primers used for RT-qPCR were synthesized by BGI company. Primers sequences were as followed: Rat: LAP3 (Forward: GACACCAACCAGATTTGCCG; Reverse: GGTGCTTCAGTTGCATTGGG); β-Actin (Forward: ACCCTAAGGCCAACCGTGAA; Reverse: GTGGTACGACCAGAGGCATAC); Homo: LAP3 (Forward: AGCCAAAAACGGGAAGACCA; Reverse: CTGTTTCAATGCTGGCCTCG); β-Actin (Forward: AAGGATTCCTATGTGGGCGAC; Reverse: CGTACAGGGATAGCACAGCC).

### Immunohistochemistry and cell dual immunofluorescence staining

To localized the LAP3 expression, paraffin-embedded E3 liver tissues were applied for immunohistochemistry evaluation as previously described [41]. For the cell dual immunofluorescence staining was carried out following previously described [37, 38]. The LAP3 primary antibodies were purchased from Santa Cruz (Santa Cruz Biotechnology, sc-376270, China). Thereafter, both immunofluorescence pictures and IHC staining pictures were taken under an Olympus (Japan) microscope. The immunofluorescence pictures were merged and quantified with Image-Pro Plus 6.0 software (Media Cybernetics, Bethesda, MD, USA). IHC staining pictures were quantified with Image-Pro Plus 6.0 software (Media Cybernetics, Bethesda, MD, USA).

### RNA interference

Three Small-interfering RNA (siRNA) targeting on LAP3 (Gene ID: NM_015907.2) gene, and negative control (NC) were designed and synthesized by Shanghai Genepharma (China). The most effective siRNA (si-LAP3) which was identified by western blotting was applied for further experiments. The sequence is as following Homo: siLAP3-1 (Forward: CCAACCAGAUUUGCUGAAATT; Reverse: UUUCAGCAAAUCUGGUUGGTT); siLAP3-2 (Forward: GGUGCCAUGGAUGUAGCUUTT; Reverse: AAGCUACAUCCAUGGCACCTT); siLAP3-3 (Forward: GCAUGUACAGCUGCAGC AUTT; Reverse: AUGCUGCAGCUGUACAUGCTT). LO2 cells were seeded in a 6-well plate (Corning Inc., Corning, NY, USA) at 30% confluence, and the cell was transfected with siRNA following the manufacturer’s protocols. Briefly, siLAP3 or siNC sequences were mixed with Lipofectamine 2000 (Invitrogen; Carlsbad; CA, USA) at final concentrations of 10 nM/ml in serum-free media, and incubated with cells. After 6 h of transfection, we replaced serum-free medium with 10% FBS RPIM 1640 medium, and harvested cells after 48 hours of transfection.

### Cell viability analysis

Cell viability was evaluated using the CCK-8 (Beyotime, Shanghai, China) assay. LO2 cells were seeded in 96-well plates at a density of 5 × 10^3^ cells and treated with different concentrations of bestatin (Selleck, Shanghai, China) the next day, following cultured for 1–4 days in 100 μL of 1640 medium. Then, the cells were placed at 37°C in the dark for reacting an hour after adding CCK-8 reagent to each well according to the instruction of the manufacturer, the absorbance of the density of each well was read at a wavelength of 450 nm with a microplate reader.

### Measurement of intracellular ROS levels

Intracellular reactive oxygen species (ROS) generation was assessed flow cytometry using the peroxide-sensitive fluorescent probe 2′,7′-dichlorofluorescein diacetate (DCFH-DA) (Beyotime, Shanghai, China) following instructions. After collection, cells were incubated with the DCFH-DA dye, which was diluted in serum-free 1640 medium at a proportion of 1:1,000 for 30 min at 37°C in the dark, washed twice with PBS, and then resuspended in PBS to detect the generation of intracellular ROS by flow cytometry (BD C6 Biosciences). In all experiments, 10,000 viable cells were analyzed. Data analysis was performed by using Graph prism 8.0.

### Determination of GSH and GSSG

The intracellular glutathione (GSH) and glutathione disulfide (GSSG) level was measured by GSH and GSSG Assay Kit (Beyotime; Shanghai; China) according to the kit instruction. LO2 cells were treated with 150 μM cholesterol for 0 h, 3 h, 6 h, 9 h, 12 h, 24 h respectively, harvested, and then lysed by releasing buffer on ice. Based on the protocols of the manufacturer, the standard curve of the absorbance to GSH and GSSG concentrations was measured. Then, we determined the GSH and GSSG concentration using a microplate reader at 412 nm.

### Bioinformatic analysis

The Pathway was obtained directly from the KEGG database (http://www.genome.jp/kegg/). Proteins interacting with LAP3 were retrieved from the STRING database (http://string-db.org/).

### The collection of serum samples from healthy people and patients with NAFLD

We enrolled the 80 Han Chinese serum samples from B ultrasound-proven NAFLD or BMI ≥ 24 with persistent half-year elevation of ALT and AST or TC and TG (*n* = 40), and healthy people (*n* = 40) were collected in the First Affiliated Hospital of Xi’an Jiaotong University from 2018 to 2020. We strictly excluded patients with liver disease autoimmune, infection with hepatitis viruses, genetic liver diseases and ethanol consumption above 100 g per week from our study, and the detailed clinical characteristics were presented in Table 1. The study was approved by the First Affiliated Hospital of Xi’an Jiaotong University research ethics committee and all subjects provided oral informed consent.

### Transmission electron microscope

To examine the influence of LAP3 alteration on autophagy, LO2 cells were fixed with 2% glutaraldehyde/0.1M PBS (pH 7.2) at 4°C for 2 h after the respective treatments, then post-fixed in 1% osmium tetroxide. Afterward, washed cells with PBS and then dehydrated with ascending gradient of ethanol (50%, 75%, 95%, and 100% ethanol). Subsequently, the cells were embedded in propylene oxide embedding resin. Thereafter, an LKB-V ultramicrotome (LKB, Sweden) was used to cut the resin into the ultrathin section further. And then the resins were mounted onto copper grids. Lastly, the resins were double-stained with uranyl acetate and lead citrate. The sample images were visual using an H-7650 (HITACHI, Ibaraki, Japan) transmission electron microscope.

### Statistical analysis

The difference between the two groups was analyzed with an unpaired Student’s *t*-test in this study. A one-way ANOVA was performed when comparing the differences between more than two groups. Data we expressed as the means ± SD. Pearson’s test was used for correlation analysis. Data were analyzed using GraphPad Prism 8.0 software (GraphPad Prism, CA, USA) and a ^*^p < 0.05, ^**^p < 0.01, and ^***^p < 0.001 were considered the presence of statistical significance between experimental groups.
